# PMAT: an efficient plant mitogenome assembly toolkit using low-coverage HiFi sequencing data

**DOI:** 10.1093/hr/uhae023

**Published:** 2024-01-26

**Authors:** Changwei Bi, Fei Shen, Fuchuan Han, Yanshu Qu, Jing Hou, Kewang Xu, Li-an Xu, Wenchuang He, Zhiqiang Wu, Tongming Yin

**Affiliations:** State Key Laboratory of Tree Genetics and Breeding, Co-Innovation Center for Sustainable Forestry in Southern China, Key Laboratory of Tree Genetics and Biotechnology of Educational Department of China, Key Laboratory of Tree Genetics and Silvicultural Sciences of Jiangsu Province, Nanjing Forestry University, Nanjing 210037, China; Department of artificial intelligence, College of Information Science and Technology, College of Information Science and Technology, Nanjing Forestry University, Nanjing 210037, China; Institute of Biotechnology, Beijing Academy of Agriculture and Forestry Sciences, Beijing 100097, China; Research Institute of Subtropical Forestry, Chinese Academy of Forestry, Hangzhou 311400, China; State Key Laboratory of Tree Genetics and Breeding, Co-Innovation Center for Sustainable Forestry in Southern China, Key Laboratory of Tree Genetics and Biotechnology of Educational Department of China, Key Laboratory of Tree Genetics and Silvicultural Sciences of Jiangsu Province, Nanjing Forestry University, Nanjing 210037, China; State Key Laboratory of Tree Genetics and Breeding, Co-Innovation Center for Sustainable Forestry in Southern China, Key Laboratory of Tree Genetics and Biotechnology of Educational Department of China, Key Laboratory of Tree Genetics and Silvicultural Sciences of Jiangsu Province, Nanjing Forestry University, Nanjing 210037, China; State Key Laboratory of Tree Genetics and Breeding, Co-Innovation Center for Sustainable Forestry in Southern China, Key Laboratory of Tree Genetics and Biotechnology of Educational Department of China, Key Laboratory of Tree Genetics and Silvicultural Sciences of Jiangsu Province, Nanjing Forestry University, Nanjing 210037, China; State Key Laboratory of Tree Genetics and Breeding, Co-Innovation Center for Sustainable Forestry in Southern China, Key Laboratory of Tree Genetics and Biotechnology of Educational Department of China, Key Laboratory of Tree Genetics and Silvicultural Sciences of Jiangsu Province, Nanjing Forestry University, Nanjing 210037, China; Shenzhen Branch, Guangdong Laboratory of Lingnan Modern Agriculture, Key Laboratory of Synthetic Biology, Ministry of Agriculture and Rural Affairs, Agricultural Genomics Institute at Shenzhen, Chinese Academy of Agricultural Sciences, Shenzhen 518000, China; Shenzhen Branch, Guangdong Laboratory of Lingnan Modern Agriculture, Key Laboratory of Synthetic Biology, Ministry of Agriculture and Rural Affairs, Agricultural Genomics Institute at Shenzhen, Chinese Academy of Agricultural Sciences, Shenzhen 518000, China; State Key Laboratory of Tree Genetics and Breeding, Co-Innovation Center for Sustainable Forestry in Southern China, Key Laboratory of Tree Genetics and Biotechnology of Educational Department of China, Key Laboratory of Tree Genetics and Silvicultural Sciences of Jiangsu Province, Nanjing Forestry University, Nanjing 210037, China

## Abstract

Complete mitochondrial genomes (mitogenomes) of plants are valuable resources for nucleocytoplasmic interactions, plant evolution, and plant cytoplasmic male sterile line breeding. However, the complete assembly of plant mitogenomes is challenging due to frequent recombination events and horizontal gene transfers. Previous studies have adopted Illumina, PacBio, and Nanopore sequencing data to assemble plant mitogenomes, but the poor assembly completeness, low sequencing accuracy, and high cost limit the sampling capacity. Here, we present an efficient assembly toolkit (PMAT) for *de novo* assembly of plant mitogenomes using low-coverage HiFi sequencing data. PMAT has been applied to the *de novo* assembly of 13 broadly representative plant mitogenomes, outperforming existing organelle genome assemblers in terms of assembly accuracy and completeness. By evaluating the assembly of plant mitogenomes from different sequencing data, it was confirmed that PMAT only requires 1× HiFi sequencing data to obtain a complete plant mitogenome. The source code for PMAT is available at https://github.com/bichangwei/PMAT. The developed PMAT toolkit will indeed accelerate the understanding of evolutionary variation and breeding application of plant mitogenomes.

## Introduction

Mitochondria are double-membrane-bound organelles found in most eukaryotes. As the site of oxidative energy metabolism, mitochondria not only supply energy for complex cellular physiological activities but are also widely involved in signaling transduction, cell differentiation, cell cycle and growth regulation, and cell death [1–4]. However, the extensive genome variation of the plant mitochondrial genome hinders our understanding of its evolutionary meaning.

Plant mitochondria differ substantially from those of other eukaryotes in several aspects [5, 6], including greater gene content, variable genome size, complex genome structure, and ability to integrate exogenous DNAs. Frequent recombination mediated by repeats and horizontal gene transfer (HGT) is the major driver of changes in the size and structure of the mitochondrial genome (mitogenome) [7–10] and the main factor affecting plant mitogenome evolution. Unlike the conserved single circular genome in animal mitochondria, the *in vivo* structure of the plant mitogenome is far more complex than a single master circle could suggest [11]. In addition to the typical circular structure, some plant species also have multi-chromosomal or even linear structures [6, 12–14]. Abundant repeats and HGTs may affect seed extension during mitogenome assembly and ultimately result in failure to obtain a complete mitogenome. To date, there are ~3000 plant nuclear genomes and ~12 000 plant chloroplast genomes, but only ~500 plant mitogenomes are publicly available at NCBI GenBank (accessed on 31 July 2023).

Currently, plant mitogenomes can be assembled either based on mitochondrial DNA (mtDNA) sequencing, which requires mitochondrial DNA isolation and purification [15–17], or directly from whole-genome sequencing (WGS) data [18–21]. However, efficiently isolating and enriching mtDNA while avoiding nuclear DNA contamination is a challenge for many studies on plants. Additionally, different species and tissues have different phenolic compounds and metabolite profiles, which can easily disrupt the integrity of mitochondrial membranes, resulting in an extremely species- and tissue-specific approach to plant mtDNA isolation [22, 23].

Although multiple methods for assembling nuclear or organelle genomes using WGS data have been applied to assemble plant mitogenomes, their assembling quality varies widely. For example, SPAdes [24], NOVOPlasty [25], and GetOrganelle [26] use Illumina short-read sequencing data for *de novo* plant mitogenome assembling. These methods could generate a relatively complete mitogenome when the assembled mitogenome lacks repeats and has a single master circular structure. But for mitogenomes with many repeats, these methods will ultimately fail to obtain a complete mitogenome due to the inability to span most repeats using short-read sequencing data. To address the issues in assembling mitochondrial repeats, SMARTdenovo [27], NextDenovo [28], Canu [29], and hifiasm [30] have utilized long-read sequencing data. However, they all directly break contigs or only extend the path with the highest read number when assembling repeat-mediated recombination and are unable to obtain more possible mitogenome conformations [18, 21, 31, 32]. Additionally, some plant mitogenomes are assembled by integrating the assembling results from Illumina and PacBio/Nanopore sequencing data [21, 33–35], which is very complicated and relies heavily on experienced manual corrections. The above assembling strategies can only acquire some conformations of the mitogenome because these assembling methods usually interrupt the contigs directly or only select the path with the most reads when encountering multiple branches caused by repeats and HGT. Many published plant mitogenomes are assembled only as a single master circular chromosome without resolving more possible mitochondrial conformations [16, 36, 37].

Recently, a plant graphical assembly tool named GSAT was developed to assemble the complex conformation of plant mitogenomes [38]. GSAT relies on Illumina sequencing data to construct the initial assembly graph firstly, and further simplify the graph using third-generation sequencing data to obtain the mitogenome pan-structural assembly graph. GSAT has been successfully applied to assemble mitogenomes of *Arabidopsis thaliana* and *Oryza sativa*. However, this method relies on Illumina data to construct the initial assembly graph, which can be seriously corrupted by large repetitive and HGT sequences, resulting in the inability to obtain the complete mitogenomes even using third-generation sequencing data.

How to capture all mitogenome conformations from plant WGS data becomes an urgent problem to be solved in plant mitogenomic and evolutionary studies. In this study, we present an efficient toolkit (PMAT) for assembling plant mitogenome pan-structure using ultra-low HiFi (high-fidelity) sequencing data without requiring mitochondrial DNA isolation and subsequent gap closure. PMAT includes a Singularity container [39] and several scripts for recruiting target mitochondrial contigs from third-generation WGS data and generating reliable plant mitogenome assembly graphs for user-friendly manual completion and correction. Using PMAT, we successfully assembled the mitogenomes of 13 plant species across the plant tree of life and evaluated the minimal sequencing data required for assembling a complete plant mitogenome. Overall, the study provides an efficient toolkit for assembling complex plant mitogenomes and important mitogenome resources for plant evolution and phylogeny.

## Results

### Functions and features of PMAT

**Figure 1:**
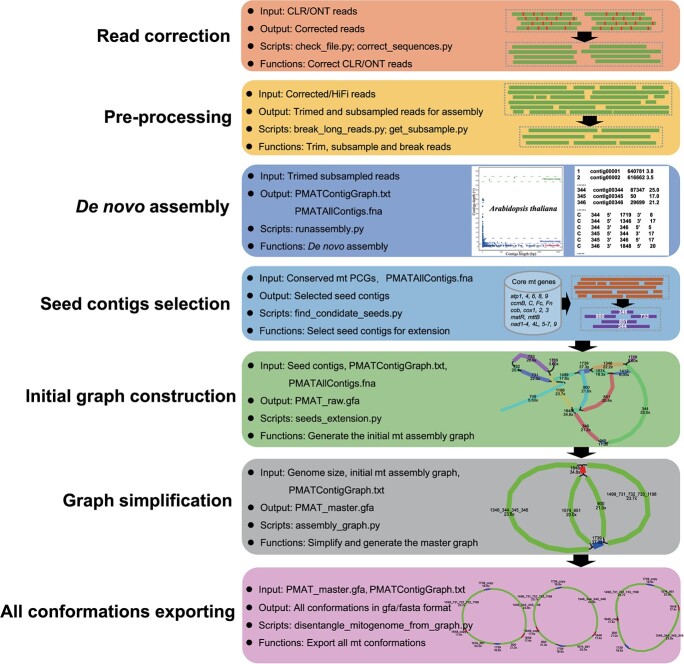
Automatic workflows of the plant mitogenome assembly toolkit. All listed Python scripts are available at https://github.com/bichangwei/PMAT.

Unlike the typical single circular structure of the chloroplast genome and the animal mitogenome, the *in vivo* structure of plant mitogenomes is far more complex than a single master circle could suggest. Obtaining the actual panoramic plant mitogenome is still considered a roadblock in plant evolutionary biology. PMAT is a new open-source toolkit for plant mitogenome assembly, which uses whole-genome CLR/ONT/HiFi sequencing data as input and outputs a complete and accurate mitogenome graph (Fig. 1). PMAT is a *de novo* graph-based assembler that can construct the pan-structural landscape of the plant mitogenome using ultra-low-coverage HiFi sequencing data. The generated panoramic plant mitogenome in GFA format can be further used to generate the master and other possible mitogenome sequences. PMAT has two modes: ‘autoMito’ and ‘graphBuild’. The former is a one-step assembler that allows users to obtain a complete mitogenome graph by simply providing the raw sequencing data and specifying the sequencing type and its nuclear genome size. If PMAT fails to generate the assembly graph in autoMito mode, users can use graphBuild mode to manually select appropriate seeds for assembly. Additionally, PMAT is also applicable to assemble chloroplast genomes with the —type pt parameter.

### Mitogenome assembly of eudicots

**Figure 2:**
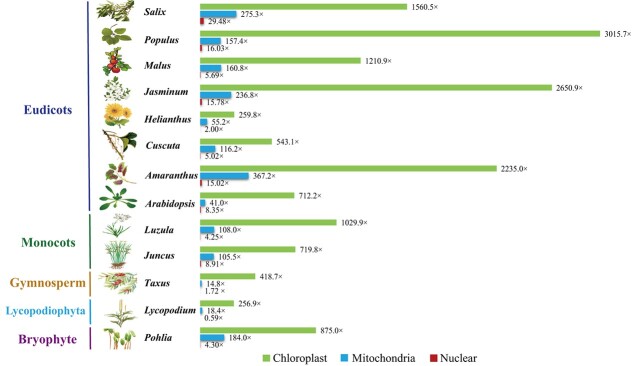
Average contig depths in chloroplast, mitochondrial, and nuclear genomes. The chloroplast, mitochondrial, and nuclear contig depths are shown in green, blue, and red, respectively.

To test the performance of PMAT, we first assembled the complete mitogenomes of eight eudicots using PMAT. Among them, four (*A. thaliana* Col-0, *Cuscuta europaea*, *Helianthus annuus* ANN1372–3, and *Malus domestica* Costard) were reassembled in this study and the other four (*Amaranthus tricolor*, *Jasminum sambac*, *Populus trichocarpa*, and *Salix wilsonii*) were *de novo* assembled for the first time (Table 1). As shown in Fig. 2, the average contig depths of chloroplast, mitochondrial, and nuclear genomes of each species were completely different, so the assembled contigs belonging to the mitogenome could be easily distinguished by their lengths and depths.

**Table 1:** Summary of all assembled mitogenomes in this study.

**Organism**	**SRA accession**	**Sequencing data (Gbp)**	**Estimated genome coverage (×)**	**NCBI released mitogenome size (bp)**	**Assembled size of this study (bp)**	**Accession number**
*Pohlia nutans*	CRR383826^a^	31.16	44.63	99 864	99 733	NC_046778^b^
*Lycopodium japonicum*	SRR24785435^c^	2.4	0.59		454 458	OR046024^d^
*Taxus chinensis*	SRR14756467^e^	17.66	1.72		469 770	OP177687^d^
*Juncus effusus*	ERR8282830^e^	22.55	100.45		519 026	OP177680^d^
*Luzula sylvatica*	ERR8705854^e^	4.74	4.25		633 359	OP177679^d^
*Arabidopsis thaliana*	CRR302668^a^	22.9	191.31	367 808	367 810	NC_037304^b^
*Amaranthus tricolor*	CRR511440^a^	25.1	53.86		382 432	OP177683-85^d^
*Cuscuta europaea*	ERR9250942^e^	25.9	26.54	406 647	406 648	BK059238^b^
*Helianthus annuus*	SRR14782853^e^	12.82	4.26	300 945	300 887	NC_023337^b^
*Jasminum sambac*	SRR17758539^e^	43.2	85.19		508 930	OP177681^d^
*Malus domestica*	ERR6939264^e^	9.39	13.35	396 947	396 949	NC_018554^b^
*Populus trichocarpa*	SRR22064349^c^	6.96	16.03		803 673	MZ826271-73^d^
*Salix wilsonii*	SRR21570388^c^	10.26	29.48		711 456	NC_064688^d^

a Downloaded from GSA.

b Publicly available at NCBI.

c Sequenced in this study.

d Submitted to NCBI in this study.

e Downloaded from SRA.

**Figure 3:**
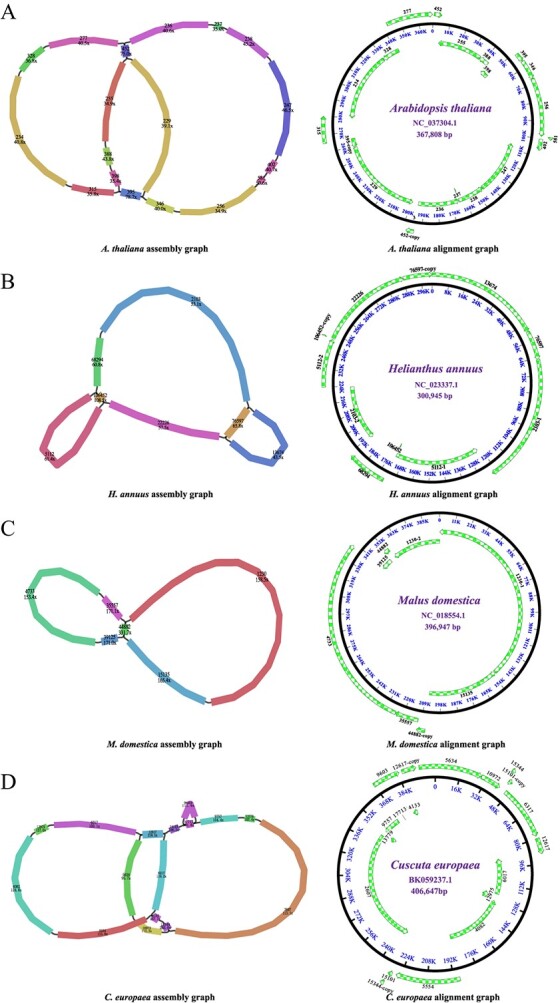
Assembly and alignment graphs of four eudicot mitogenomes. **A***Arabidopsis thaliana*. **B***Helianthus annuus*. **C***Malus domestica*. **D***Cuscuta europaea*. Green arrows outside the circle represent contigs resulting from the assembly. Each colored contig is labeled with its name and sequencing depth.

As shown in Fig. 3 and Table 1, the contigs of the four reassembled mitogenomes almost completely cover their corresponding reference mitogenomes [40–44], confirming the validity and accuracy of our assembling procedure. The *A. thaliana* Col-0 mitogenome was reassembled into a typical single circular chromosome with a length of 367 810 bp, showing only 2 bp difference from the published *A. thaliana* Col-0 mitogenome (accession number NC_037304.1, length 367 808 bp). The reassembled *H. annuus* cytoplasmic fertile (ANN1372-3) mitogenome was 300 887 bp in length, only 58 and 60 bp shorter than the other two *H. annuus* cytoplasmic fertile mitogenomes (HA412 and HA89), respectively [42, 43]. The differences in mitogenome sizes between cytoplasmic fertile and CMS lines are due to several deletions and insertions [43]. The *M. domestica* mitogenome was also reassembled into a single circular chromosome (length 396 949 bp), showing only 2 bp difference from the published *M. domestica* mitogenome (accession number NC_018554.1, length 396 947 bp) [41]. The mitogenome of *C. europaea* was reassembled into a single circular mitogenome with a length of 406 647 bp, showing only one base difference from its published mitogenome (accession number BK059238; length 406 648 bp).

The other four *de novo* assembled eudicot mitogenomes were annotated to determine whether they contained overwhelmingly conserved mitochondrial protein-coding genes (PCGs). For ease of visualization and description, we annotated only one possible conformation for each *de novo* assembled mitogenome. As shown in Supplementary Data Fig. S1, the typical single circular mitogenome of *J. sambac* (length 508 930 bp) was assembled from 20 contigs, seven of which (contigs 24 391, 24 259, 24 163, 24 162, 24 508, 19 587, and 24 380) had two copies and one (contig 24 383) had three copies (Supplementary Data Table S1). These multicopy contigs may be involved in mediating genome recombination, leading to some non-dominant conformations. The *J. sambac* mitogenome contained 42 PCGs, 3 rRNAs, and 20 tRNAs, covering all of the 24 core mitochondrial PCGs. Similarly, the *S. wilsonii* mitogenome (length 711 456 bp) was assembled from only three contigs. None of them had multiple copies (Supplementary Data Fig. S2). A total of 58 genes (33 PCGs, 3 rRNAs, and 22 tRNAs) were annotated in the *S. wilsonii* mitogenome, including all core mitochondrial PCGs. Although the mitogenomes of *A. tricolor* and *P. trichocarpa* were assembled into atypical multi-circular structures (Supplementary Data Figs S3 and S4), all core mitochondrial PCGs could be detected in them (Supplementary Data Table S2).

### Mitogenome assembly of monocots

**Figure 4:**
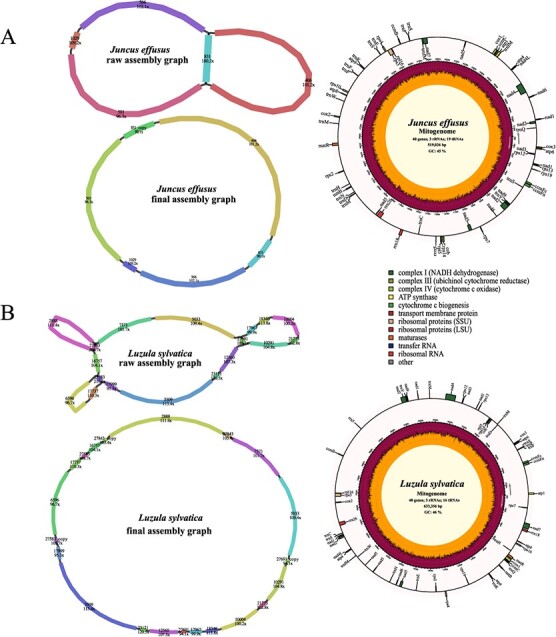
Assembly graphs and genome maps of two monocot mitogenomes. **A***Juncus effusus*. **B***Luzula sylvatica*.

We also tested the ability of our assembling procedure in two monocots: *Juncus effusus* and *Luzula sylvatica*. The *J. effusus* mitogenome was *de novo* assembled from five contigs into a 519 026-bp circular structure (Fig. 4A). It contained a pair of long repeats (contig 831) (Supplementary Data Table S1), which may be involved in mediating genome recombination. The *J. effusus* mitogenome was annotated to have 62 genes, including 3 rRNA genes, 19 tRNA genes, 16 variable PCGs, and 24 core mitochondrial PCGs (Supplementary Data Table S2). The 633 356-bp *L. sylvatica* mitogenome was assembled from 18 contigs (Fig. 4B), containing three pairs of short repeats (contigs 27 843, 27 583, and 27 691). A total of 59 genes, including 24 core mitochondrial PCGs, 16 variable PCGs, 3 rRNA genes, and 16 tRNA genes, were annotated in the *L. sylvatica* mitogenome (Supplementary Data Table S2).

### Mitogenome assembly of *Taxus chinensis*, *Lycopodium japonicum*, and *Pohlia nutans*

**Figure 5:**
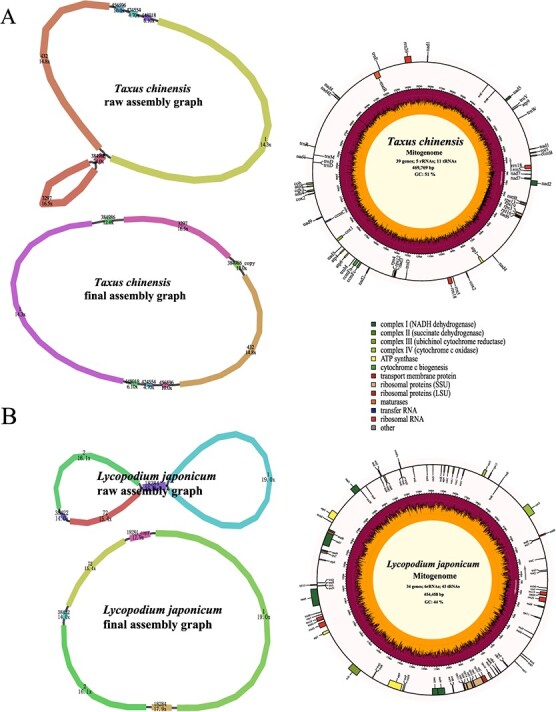
Assembly graphs and genome maps of *Taxus chinensis* (**A**) and *Lycopodium japonicum* (**B**) mitogenomes.

Compared with the heavily sequenced angiosperm clade, the gymnosperm mitogenomes were limited in number. To test the performance of PMAT in gymnosperms, a total of 17.66 Gb of whole-genome HiFi sequencing data of *Taxus chinensis* was downloaded from the NCBI Sequence Read Archive (SRA; SRR14756467) [45], with only 1.72× coverage of its nuclear genome (Table 1). Using PMAT, the *T. chinensis* mitogenome was assembled from seven contigs, containing a pair of repeats (contig 384 986) (Fig. 5A, Supplementary Data Table S1). The *T. chinensis* mitogenome was generated after decoding the raw assembly graph based on the copy number of each contig using Bandage. It was 469 769 bp in length and contained all 24 core PCGs and 15 other variable PCGs but lacked several common tRNA genes, which has been reported in two other published *Taxus* mitogenomes (*T. cuspidata* and *T. wallichiana*) [46].

Using the PacBio Revio sequencing platform, we sequenced and *de novo* assembled the mitogenome of *Lycopodium japonicum* into a typical single circular chromosome with only 0.59× HiFi WGS data (Fig. 5B). The *L. japonicum* mitogenome was annotated to contain 83 genes, including 34 PCGs, 6 rRNAs, and 43 tRNAs (Supplementary Data Table S2). Some plant core mitochondrial PCGs (*atp8*, *ccmB*, *ccmC*, *ccmFc*, *ccmFn*, *matR*, and *nad7*) could not be detected in the *L. japonicum* mitogenome, which is a very common phenomenon [47, 48].

We also reassembled the mitogenome of a bryophyte named *Pohlia nutans*. To validate the assembly accuracy, the reassembled contigs of the *P. nutans* mitogenome were mapped to its corresponding reference mitogenome (NC_046778.1) [49]. As shown in Supplementary Data Fig S5, the *P. nutans* mitogenome was reassembled into a 99 733-bp long single circular chromosome from 18 contigs. The reassembled mitogenome covered 99.87% of its reference mitogenome (99 864 bp), showing 131 bp deletions in the reference mitogenome.

### Comparison of mitogenome assemblies from purified mtDNA and whole-genome sequencing data

**Figure 6:**
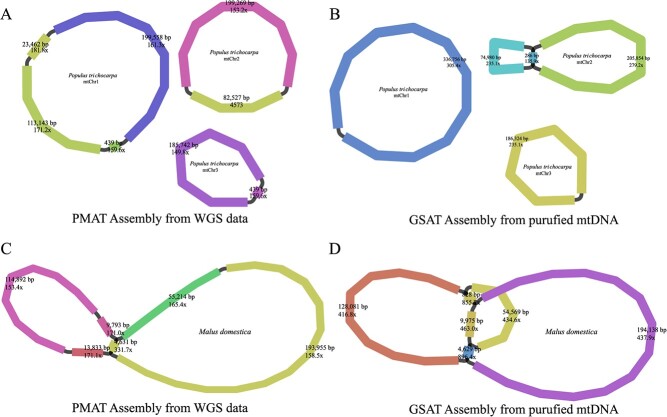
Mitogenome assembly graphs of *Populus trichocarpa* (**A**, **B**) and *Malus domestica* (**C**, **D**) were generated from PMAT (**A**, **C**) and GSAT (**B**, **D**).

To validate the assembly accuracy of PMAT, we sequenced two purified mtDNAs of *P. trichocarpa* and *M. domestica* using the Illumina NovoSeq 6000 platform. The sequencing data of *P. trichocarpa* and *M. domestica* have been submitted to the NCBI SRA repository under the accession numbers SRR24785916 and SRR24789033, respectively. Using GSAT, the purified mitogenome of *P. trichocarpa* was assembled into three circular chromosomes with a total length of 804 486 bp, showing ~99.99% similarity to the assembly graphs generated from PMAT (Fig. 6 and Supplementary Data Table S3). However, PMAT lost a repeat-mediated conformation (repeat length 286 bp) on the mtChr2 of *P. trichocarpa*. For the *M. domestica* mitogenome, the assembly graphs from PMAT (Fig. 6C) and purified mtDNA (Fig. 6D) show >99.8% similarity, but PMAT also lost a repeat-mediated (repeat length 828 bp) conformation.

### Minimal sequencing data for mitogenome assembly

After completing the assembly of mitogenomes of 13 plant species, we conducted a comprehensive simulation study to assess the minimum sequencing data required for achieving a complete mitogenome assembly using PMAT. As shown in Table 2, we were able to assemble the complete mitogenomes of *P. nutans*, *J. effusus*, *A. tricolor*, *M. domestica*, *P. trichocarpa*, and *S. wilsonii* using only 200 Mb of whole-genome HiFi sequencing data. By contrast, a minimum of 500 Mb sequencing data was required to assemble the complete mitogenomes of *A. thaliana* and *J. sambac*, while *L. japonicum*, *L. sylvatica*, *H. annuus*, and *T. chinensis* required at least 1, 1, 2, and 10 Gb of sequencing data, respectively. With increasing sequencing data the number of assembled contigs increased, leading to more complex mitochondrial assembly graphs. Strikingly, as shown in Supplementary Data Fig S6 and Table 2, the *J. sambac* mitogenome was assembled into two separate master graphs when using fewer sequencing data (500 Mb and 1 Gb) but assembled into a single master graph when using more sequencing data (8 Gb).

**Table 2:** Evaluation of minimal sequencing data required for complete mitogenome assembly.

**Organism**	**Order**	**Sampled data (Gb)**	**Genome depth (×)**	**Mitogenome depth (×)**	**Assembled contigs**	**Assembled size (bp)**	**Elapsed time (min)**	**Memory usage (Gb)**
*Pohlia nutans*	Bryales	0.2	0.29	14	1	99 735	3.4	31.4
		1	1.43	66.4	1	99 734	19	31.8
		3	4.3	184	18	99 733	36.1	32.2
*Lycopodium japonicum*	Lycopodiaceae	0.5	0.12					
		1	0.25	10.9	3	454 420	27.1	6.7
		2.4	0.59	18.4	5	454 458	221.3	57.6
*Taxus chinensis*	Pinales	5	0.49					
		10	0.97	9.3	9	469 834	231.7	38.9
		17.66	1.72	14.8	8	469 770	462.1	63.4
*Juncus effusus*	Poales	0.2	0.89	10.6	7	519 033	6.5	22.1
		1	4.45	52.2	6	519 027	21.4	34.1
		2	8.91	105.5	5	519 026	35.8	36.9
*Luzula sylvatica*	Poales	0.5	0.45					
		1	0.9	27.2	12	633 356	41.1	27.6
		4.74	4.25	108	18	633 359	125.4	51.6
*Arabidopsis thaliana*	Brassicales	0.2	1.67					
		0.5	4.78	23.1	14	367 809	9	21.4
		2	16.7	82.7	29	367 810	33.5	23.1
*Amaranthus tricolor*	Caryophyllales	0.2	0.43	9.4	4	382 400	7.1	25.9
		1	2.15	51.2	4	382 383	30.5	32.1
		7	15.02	367.2	21	382 432	208.8	51.9
*Cuscuta europaea*	Solanales	1	1.03	23.3	7	406 648	49.5	31.7
		2	2.06	47.9	13	406 647	61.3	42.9
		5	5.12	116.2	16	406 648	158.1	64.1
*Helianthus annuus*	Asterales	1	0.33					
		2	0.66	19.4	5	300 896	31.6	15.1
		6	2	55.2	7	300 887	292.2	38.7
*Jasminum sambac*	Lamiales	0.2	0.39					
		0.5	0.99	17.1	4	508 933	10.6	42.1
		8	15.78	236.8	20	508 930	413.1	72.0
*Malus domestica*	Rosales	0.2	0.28	7.6	1	396 942	8.5	9.4
		1	1.42	37.8	3	396 949	21.2	9.9
		4	5.69	160.8	6	396 949	86.5	10.2
*Populus trichocarpa*	Malpighiales	0.2	0.46	4.9	10	804 125	3.5	7.1
		1	2.3	28.2	3	803 751	25.7	33.3
		6.96	16.03	157.4	7	804 579	106.5	46.8
*Salix wilsonii*	Malpighiales	0.2	0.57	6.7	1	709 751	5.9	24.8
		1	2.87	31.1	1	711 454	23.1	34.0
		10.26	29.48	275.3	3	711 458	106.3	47.8

## Discussion

The development of sequencing technologies has promoted the study of plant mitochondrial genomics, providing important genomic resources for understanding the inheritance, structure, evolution, and function of mitochondria. Plant mitogenomes contain numerous repeats and HGT sequences, which may interfere with sequence elongation during mitogenome assembling [6, 50–54]. Therefore, it is difficult to obtain a complete mitogenome using conventional assembly methods. Many released plant mitogenomes are composed of only a single master circular chromosome [16, 36, 37] without more possible mitochondrial conformations [11, 55], which seriously hinders the development of research on plant mitochondrial structural and functional genomics. Therefore, there is an urgent need to develop an effective assembling strategy that does not require separating mitochondrial DNA from nuclear DNA to directly obtain all configurations of the mitogenome from plant WGS data.

In this study, we developed PMAT for assembling plant mitochondrial multi-conformations from whole-genome HiFi sequencing data without isolating mitochondrial DNA. By taking advantage of highly accurate long-read sequencing data, PMAT can construct multi-conformation mitogenome assembly graphs based on differences in mitochondrial, chloroplast, and nuclear genome copy numbers. Using PMAT, we successfully assembled 13 plant mitogenomes across the plant tree of life. Eight were *de novo* assembled and annotated for the first time (Table 1). Of the *de novo* assembled mitogenomes, *A. tricolor* and *P. trichocarpa* (sect. *Tacamahaca*) mitogenomes were assembled into three circular chromosomes (Supplementary Data Figs S3 and S4), while the others were assembled into typical single master circular structures. A multi-chromosomal mitogenome has been found in many species, such as *Psilotum nudum* [17], *Silene conica* [56], *Solanum tuberosum* [14], and *Actinidia chinensis* [16], but one was detected in the genus *Amaranthus* for the first time. In genus *Populus*, the mitogenomes of *P. simonii* (sect. *Tacamahaca*) and *P. deltoides* (sect. *Aegiros*) were also assembled into three circular chromosomes, while the other mitogenomes in sect. *Populus* were assembled into typical single master circular structures [8, 19]. Investigating the structural variations of different *Populus* mitogenomes is conducive to elucidating the mechanism of structural diversity formation in plant mitogenomes. The newly assembled mitogenomes in this study will provide important genomic resources for plant phylogeny, plant resource conservation, and exploitation.

Previous studies have assembled some plant mitogenomes by integrating Illumina and PacBio CLR/Nanopore WGS data. They constructed the raw assembly graph based on Illumina data using SPAdes [24], GetOrganelle [26], GSAT [38], or NovoPlasty [25] and generated the final assembly graph using PacBio or Nanopore long reads to fill the gap or solve the repeat regions [21]. However, these approaches are only effective for mitogenomes with small nuclear genomes and structurally simple mitochondrial genomes. When assembling mitogenomes with large nuclear genomes, such as *Ginkgo*, Pinales, and *Pinus* mitogenomes, it is extremely costly and time-consuming. Recently, a new Python workflow (MitoHiFi) was developed to assemble mitogenomes from PacBio HiFi data [57]. MitoHiFi needs a closely related mitogenome as reference genome, and extracts mitochondrial reads to assemble the final mitogenome. It has been widely applied to assemble the mitogenomes for a wide range of species in metazoans, but MitoHiFi was not optimized to assemble plant mitogenomes. The significant variations in size, gene content, and repeat composition observed in plant mitogenomes will prevent MitoHiFi from obtaining accurate and complete mitogenomes. In this study, PMAT takes advantage of HiFi long-read sequencing data to span most repeats to obtain complete mitogenome sequences. The PacBio CLR and ONT sequencing reads can also be used to assemble the plant mitogenomes in PMAT, but they need to be corrected prior to the initial assembling to generate high-fidelity reads. We also evaluated the minimal sequencing data required for plant complete mitogenome assembly (Table 2), and the results showed that ~200–500 Mb reads could be enough for most land plant mitogenomes. The minimal sequencing data for a complete mitogenome assembly varies widely between species, as mitogenome copy numbers can differ greatly in all plant cells (Fig. 2) [58]. The minimal sequencing depths range from 0.28× in *M. domestica* to 1.03× in *C. europaea* (Table 2). Therefore, HiFi sequencing with at least 1× coverage of the nuclear genome is recommended to obtain a complete plant mitogenome. Additionally, the benchmarking results of PMAT and hifiasm showed that PMAT could obtain more accurate mitogenome conformations with fewer HiFi sequencing data, time, and memory (Table 2 and Supplementary Data Table S4). Overall, PMAT is efficient and cost-effective for mitogenome assembly with a large nuclear genome size and can be widely applied to mitogenome studies in plant populations.

Unlike the conserved quadripartite circular structure of plant chloroplast genomes, plant mitogenomes often have multiple alternative conformations due to repetitive sequences [36, 59, 60]. Our assembly procedure can generate a raw assembly graph by recording the depth and connections of each contig. After removing false links and branches from the raw assembly graph, a simplified master assembly graph can be generated and used in Bandage to export all possible distinctive mitogenome conformations. Additionally, for most plants the number of contigs increases with sequencing data, resulting in a more complex mitochondrial assembly graph (Table 2). Therefore, using fewer HiFi sequencing data (depth: 1–3× of the nuclear genome) is a better way to get a complete mitogenome without focusing on its complex and dynamic conformations. Some extremely rare mitochondrial conformations may not be captured with fewer sequencing data, and more sequencing data may be required if the user plans to capture more possible conformations. It should be noted that our assembly strategy may lose some of the real mitochondrial conformations due to the low sequencing depth. However, the current effective PMAT toolkit has covered more of the mitochondrial conformations than other tools and includes the full gene content.

## Materials and methods

### Whole-genome sequencing and public data download

This study assembled the mitogenomes of 13 plant species, including one bryophyte (*P. nutans*), one lycophyte (*L. japonicum*), one gymnosperm (*T. chinensis*), two monocots (*J. effusus* and *L. sylvatica*), and seven eudicots (*A. thaliana* Col-0, *A. tricolor*, *H. annuus* ANN1372–3, *J. sambac*, *M. domestica* Costard, *P. trichocarpa*, and *S. wilsonii*). The HiFi data of *L. japonicum*, *P. trichocarpa*, and *S. wilsonii* were sequenced for the first time in this study, while the other data were downloaded from the Genome Sequence Archive (GSA) and NCBI Sequence Read Archive (SRA) databases (Table 1).

Fresh *P. trichocarpa* and *S. wilsonii* leaves were collected from the campus of Nanjing Forestry University, Nanjing, China (32°04′41″ N, 118°48′23″ E) and stored at −80°C for future use. Fresh leaves of *L. japonicum* were collected from Kunming Institute of Botany, Kunming, China (25°07′05″ N, 102°44′15″ E). Genomic DNA was extracted using the Hi-DNAsecure Plant Kit (Tiangen DP350). The purity and integrity were checked by agarose gel electrophoresis and a Nanodrop 2000 ultraviolet spectrophotometer (ThermoFisher). Then, high-integrity genomic DNA was used to construct sequencing libraries using SMRTbell Express Template Prep Kit 2.0 (PacBio Biosciences, CA, USA). The sequencing libraries of *P. trichocarpa* and *S. wilsonii* were sequenced on the PacBio Sequel II platform using the Circular Consensus Sequence (CCS) mode, while the library of *L. japonicum* was sequenced on the PacBio Revio platform.

### Workflow of PMAT

The workflow of PMAT is shown in Fig. 1, and includes the following six main steps.

#### Step 1. Read correction

PMAT uses long sequencing reads (CLR/ONT/HiFi) to build the initial assembly graph, but the more accurate HiFi sequencing reads are highly recommended in PMAT because the sequencing error in CLR and ONT can affect identification efficiency during assembly. In the case of CLR or ONT sequencing data, PMAT uses correct_sequences.py (under the directory ‘modules’) to correct them with NextDenovo (default option) or the Canu correction module.

#### Step 2. Data preprocessing and de novo assembly

Newbler software was originally developed to assemble Roche 454 sequencing data based on the Overlap-Layout-Consensus algorithm [61, 62]. The assembly results of Newbler retain all repeat-mediated branching structures and record their read depths, thus presenting clear advantages when assembling plant mitogenomes with multi-conformations [8, 63, 64]. PMAT breaks the long HiFi or corrected reads (>30 kb) into more shorter reads with different step lengths (default: 20 kb) using break_long_reads.py and further assembles them with the assembly software Newbler, which is packaged in a container called runAssembly.sif (under directory ‘container’). In the PMAT autoMito mode, increasing the minimum overlap length (−ml) from 90 to 98 and the minimum overlap identity (−mi) from 40 to 100 or higher may yield better results.

#### Step 3. Selecting seed contigs for extension

The file named PMATContigGraph.txt in the assembly_result directory records all the read depths and the relatedness of contigs, which can be used to build the assembly graph [64]. Because the initial mitogenome contig graph is mixed with other repeat-containing nuclear or chloroplast contigs, it was necessary to select appropriate seed contigs to capture more mitochondrial-like contigs.

To generate the candidate seed contigs, PMAT takes the file named PMATAllContigs.fna in the assembly directory as the queries, and conducts the BLASTn [65] search against a local database (under directory Conserved_PCGs_db), which is constructed using 24 conserved plant mitochondrial PCGs (*atp1*, *atp4*, *atp6*, *atp8*, *atp9*, *ccmB*, *ccmC*, *ccmFc*, *ccmFn*, *cob*, *cox1*, *cox2*, *cox3*, *matR*, *mttB*, *nad1*, *nad2*, *nad3*, *nad4*, *nad4L*, *nad5*, *nad6*, *nad7*, and *nad9*) from the representative mitogenomes of *A. thaliana*, *Brassica napus*, *Glycine max*, *Populus alba*, *Nicotiana tabacum*, *M. domestica*, *O. sativa*, *Sorghum bicolor*, *Tripsacum dactyloides*, *Triticum aestivum*, *Zea mays*, *Ginkgo biloba*, *Cycas taitungensis*, *Marchantia polymorpha*, and *Orthotrichum callistomum*. The BLASTn result is then processed by find_condidate_seeds.py to select the candidate seed contigs (lengths >500 bp; identity >85%; coverage >90%) for subsequent extension.

#### Step 4. Extending the seed contigs and constructing the initial assembly graph

PMAT then uses the Breadth First Search (BFS) algorithm embedded in seeds_extension.py to extend the seed contigs to recruit all target mitochondrial contigs according to the contig connections in the file PMATContigGraph.txt. It starts with the selected seed contigs and traverses all contigs at the current depth level before moving on to the contigs at the next depth level till all the contigs are visited. During the extension process, PMAT does not filter any contigs and connections to obtain more comprehensive mitogenome assembly graphs. The captured contigs and their connections are then fed to assembly_graph.py to generate the initial assembly graph (GFA format) based on the file PMATAllContigs.fna. In the assembly graph, contigs are the nodes and reads spanning between them (starting in one contig and continuing or ending in another) are the paths.

#### Step 5. Simplifying the mitogenome assembly graph

Since the initial mitogenome assembly graph is mixed with some chloroplast or nuclear contigs, PMAT then uses assembly_graph.py to remove the full-path chloroplast and nuclear contigs from the graph based on different contig depths of the same organelle genome, which are generally proportional to their copy numbers. In PMAT autoMito mode, the default filtering depth for filtration is inferred from the genome size (option —genomesize), while in graphBuild mode the filtering depth is determined by both genome size and the input data size (option —readsize) for assembly. Contigs will be filtered out if their depths are greater than twice the average depth of their nuclear genomes.

Considering frequent fragment transfers between chloroplast and mitochondrial genomes, PMAT reserves some essential chloroplast-like contigs for further analysis. These reserved chloroplast-like contigs can be detected by the lower depths of their connected contigs. However, some full-path chloroplast-like contigs will be removed from the assembly graph when both ends of them are connected to chloroplast contigs with higher depths. Based on the assumption that the topology of the mitogenome should be represented as a single circular or linear molecule [26], PMAT removes some tip contigs from the assembly graph using assembly_graph.py. Tip contigs are defined as contigs that connect neither to any other contigs in the assembly graph nor to themselves as circular [26]. Additionally, a path will be removed from the assembly graph if its depth is less than one-fifth of the contigs connected to it at both ends. PMAT also provides the option —minLink for the user to remove false paths directly in both autoMito and graphBuild mode. To compensate for possible shortcomings in the simplification process, PMAT provides the users with a raw assembly graph in GFA format for manual disentanglement in Bandage [66].

#### Step 6. Exporting all possible conformations and manual completion

then uses the simplified assembly graph (PMAT_master.gfa) and contig label information (PMATContigGraph.txt) to further export all possible conformation(s) into GFA and FASTA file(s). Firstly, PMAT takes all mitochondrial-like contigs (PMAT_master.gfa) as the queries, and conducts the BLASTn search against the conserved PCG database. Next, all BLASTn hit contigs are sorted by depth to calculate their median, which is further used to remove ‘noisy’ and non-target contigs. Then, PMAT chooses the largest single-copy contig as the starting point to exhaustively search for all possible paths. The single- and multi-copy contigs are determined by their depths and connections. Finally, each mitogenome conformation would be exported as an independent FASTA file. When the master graph cannot be solved as a circular path or is too complicated (many repeats) to be solved, PMAT will conservatively export the final conformations. At this point, it is recommended to visualize the simplified assembly graph (PMAT_master.gfa) and manually remove noisy and non-target contigs using Bandage [66]. After removing full-path chloroplast contigs and tip contigs, the revised assembly graph can be manually disentangled based on the copy number of each contig. All possible distinctive path(s) can be exported as FASTA file(s) after merging all possible nodes using Bandage. Each path represents a possible conformation of the target mitogenome.

### Mitogenome validation and annotation

For the assembly validation of four publicly available mitogenomes, their reference mitogenomes were downloaded from the NCBI nucleotide database. As shown in Table 1, the downloaded references were *P. nutans* (NC_046778.1), *A. thaliana* Col-0 (NC_037304.1), *C. europaea* (BK059238), *H. annuus* ANN1372-3 (NC_023337.1), and *M. domestica* Costard (NC_018554.1). All mitochondrial contigs involved in the assembly were mapped onto their corresponding reference mitogenomes using MacVector v18.2.5 (https://macvector.com/) with the Align to Reference module. Some repeat contigs with double or triple sequencing depths were copied before aligning to the reference mitogenomes. The assembled mitogenomes were considered complete if they covered >95% of the publicly available reference mitogenomes.

Although PMAT can generate complete mitogenomes from WGS data without mtDNA isolation, it may lose some critical mitogenome information. To further validate the assembly accuracy of PMAT, we isolated the purified mtDNA from the callus of *M. domestica* and *P. trichocarpa*, and sequenced them on the Illumina NovaSeq6000 platform. The mitogenomes of *M. domestica* and *P. trichocarpa* mtDNA were assembled using GSAT based on the sequencing data from their purified mtDNA [38].

The other unreleased mitogenomes were annotated using GeSeq [67] and MITOFY [63]. The putative PCGs were manually checked and adjusted by referring to other evolutionarily similar plant mitogenomes using BLASTN [65]. tRNA and rRNA genes were confirmed using tRNAscan-SE v1.21 [68]. The annotation of PCGs, tRNA genes, and rRNA genes was integrated using MacVector v18.2.5.

### Evaluation of minimal sequencing data for plant mitogenome assembling

Before undertaking large-scale WGS projects, it is necessary to evaluate the minimal sequencing data required for mitogenome assembling. To determine the minimal sequencing data for plant mitogenome assembling, we randomly sampled a fraction of the total sequencing data (default: 200 Mb, 500 Mb, and 1 Gb) using the Seqtk ‘sample’ module (https://github.com/lh3/seqtk). For species with large genomes, the default sampled data were insufficient. Therefore, we randomly sampled 1, 2, and 3 Gb of data for *H. annuus* (genome size 3.01 Gb) mitogenome assembling and 5 and 10 Gb of data for *T. chinensis* (genome size 10.24 Gb) mitogenome assembling. Table 2 shows the subsampled sequencing data and the estimated mitogenome sequencing depth. The mitogenome was considered complete at the defined sequencing depth when the assembly graph was circularized and covered the entire reference mitogenome. Otherwise, more sequencing data were needed to be resampled for assembling.

## Acknowledgements

The work was supported by the National Key Research and Development Plan of China (2021YFD2200202) and the Key Research and Development Project of Jiangsu Province, China (BE2021366). The work was also supported by the Natural Science Foundation of Jiangsu Province (BK20220414), the Natural Science Foundation of the Higher Education Institutions of Jiangsu Province (22KJB220003), the National Natural Science Foundation of China (31901331), and the Innovation Program of Chinese Academy of Agricultural Sciences. We thank Dr Weishu Fan from Kunming Institute of Botany for providing the sample of *Lycopodium japonicum*.

## Author contributions

C.B., F.S., and F.H. planned and designed the research. C.B., F.H., Y.Q., and W.H. wrote the code and processed the data. J.H., K.X., and L.X. provided the materials used in this study. C.B. and F.H. analyzed the data and prepared the figures. C.B. wrote the initial version of the manuscript; F.S., Z.W., and T.Y. revised and provided comments. Z.W. and T.Y. supervised the project.

## Data availability

The PacBio HiFi sequencing data of *L. japonicum*, *P. trichocarpa*, and *S. wilsonii* have been submitted to the NCBI Sequence Read Archive (SRA) repository under SRR24785435, SRR3204721, and SRR21570388, respectively. The Illumina sequencing data of the purified mtDNA of *P. trichocarpa* and *M. domestica* have been deposited in the SRA repository under SRR24785916 and SRR24789033, respectively. Other datasets used in this study were downloaded from GSA and SRA repositories with the accession numbers listed in Table 1. All *de novo* assembled and annotated mitogenomes have been submitted to the NCBI Nucleotide Database (https://www.ncbi.nlm.nih.gov/nuccore/) with the accession numbers listed in Table 1. The scripts of PMAT are available at https://github.com/bichangwei/PMAT.

## Conflict of interest

The authors declare no conflicts of interest.

## Supplementary data

Supplementary data is available at *Horticulture Research* online.

## Supplementary Material

Web_Material_uhae023
