# Supplementary material for: PMAT: an efficient plant mitogenome assembly toolkit using low-coverage HiFi sequencing data
Source: Hortic Res. 2024 Jan 26;11(3):uhae023. doi: 10.1093/hr/uhae023 (PMC10925850; doi:10.1093/hr/uhae023)
Supplement: Web_Material_uhae023 [file web_material_uhae023.zip › Supplymentary Data.docx]

Supplementary Data


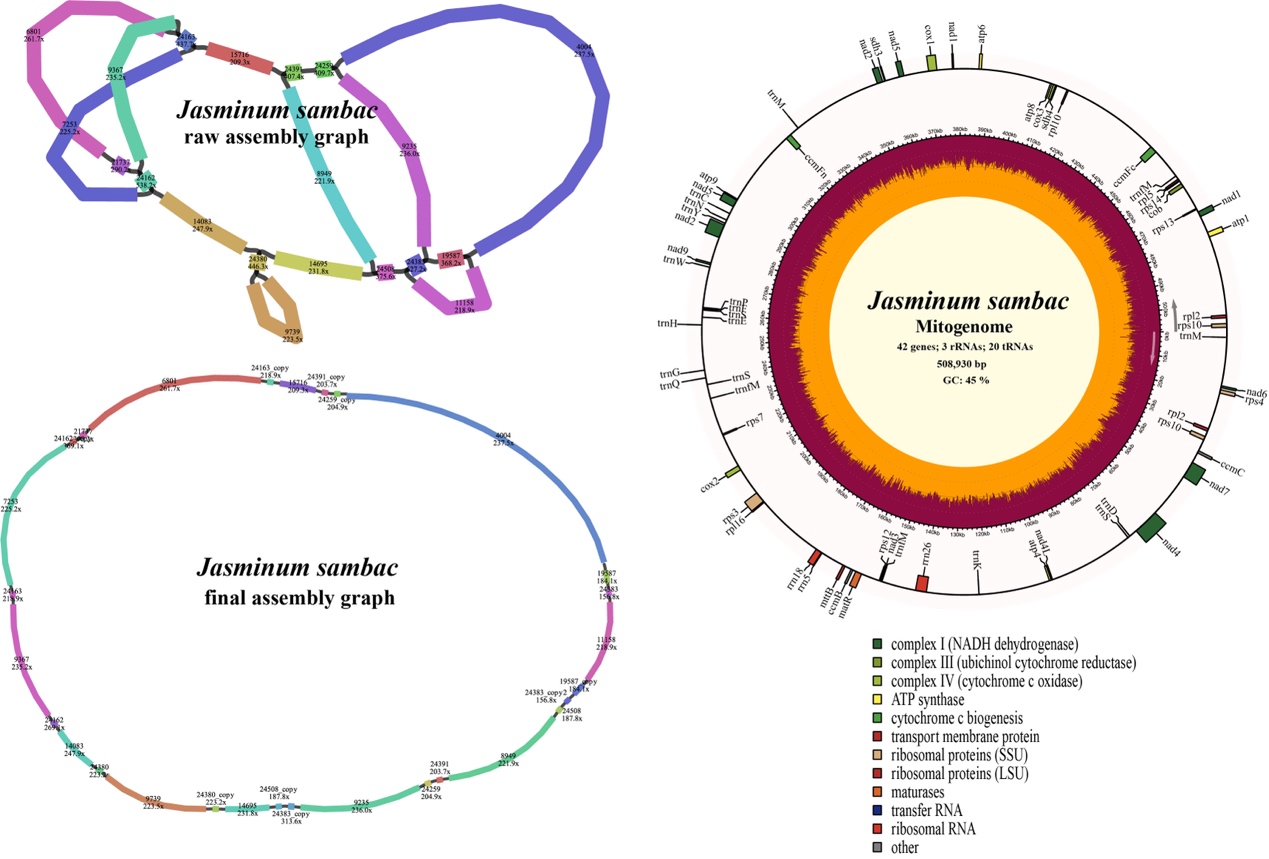


Figure S1. The assembly graphs and genome map of the *Jasminum samba* mitogenome. Each colored contig is labeled with name and sequencing depth.


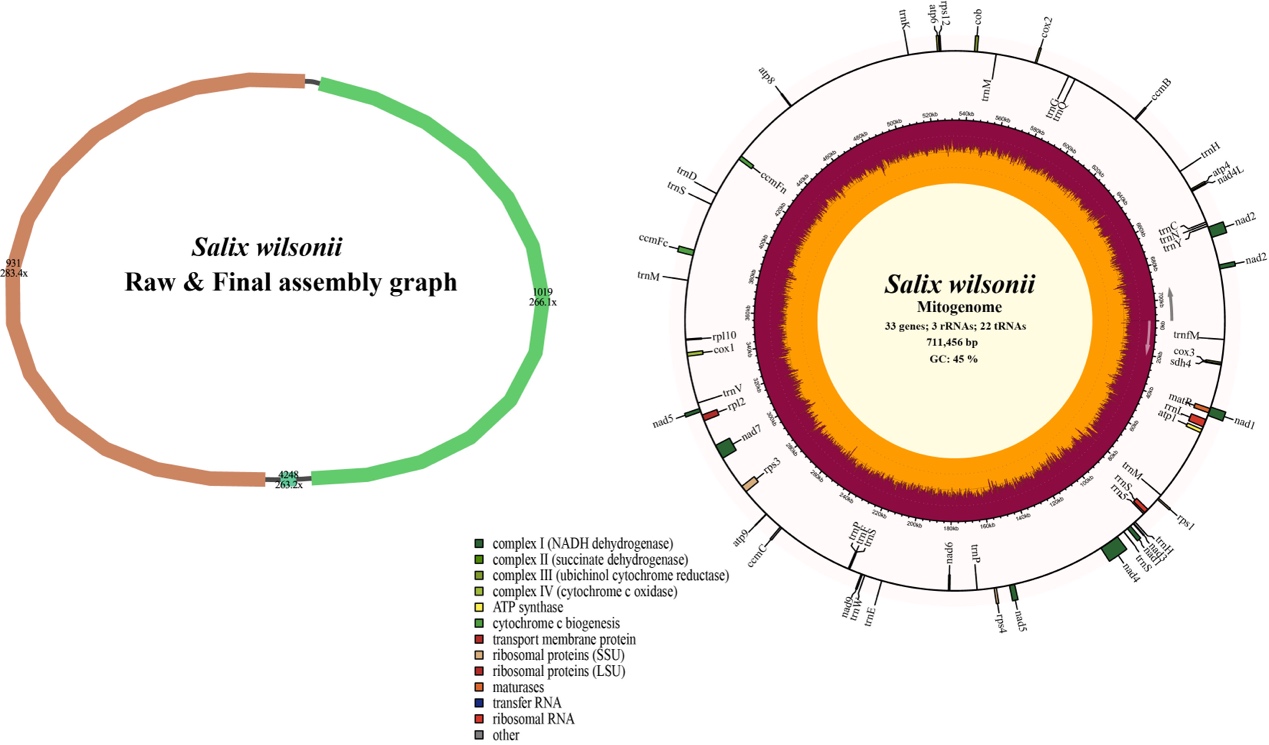


Figure S2. The assembly graph and genome map of the *Salix wilsonii* mitogenome.


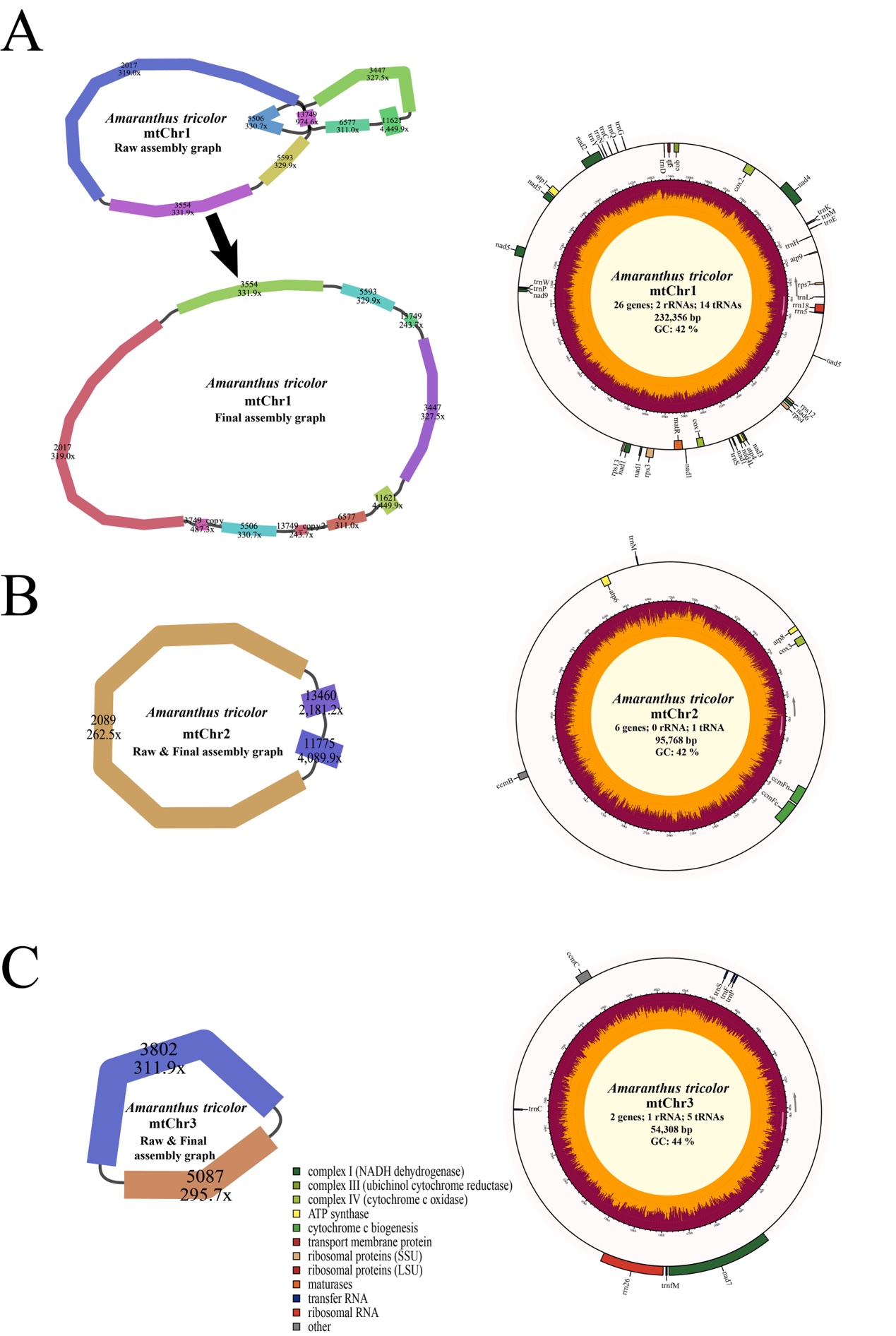


Figure S3. The assembly graphs and genome maps of the *Amaranthus tricolor* multi-circular mitogenome.


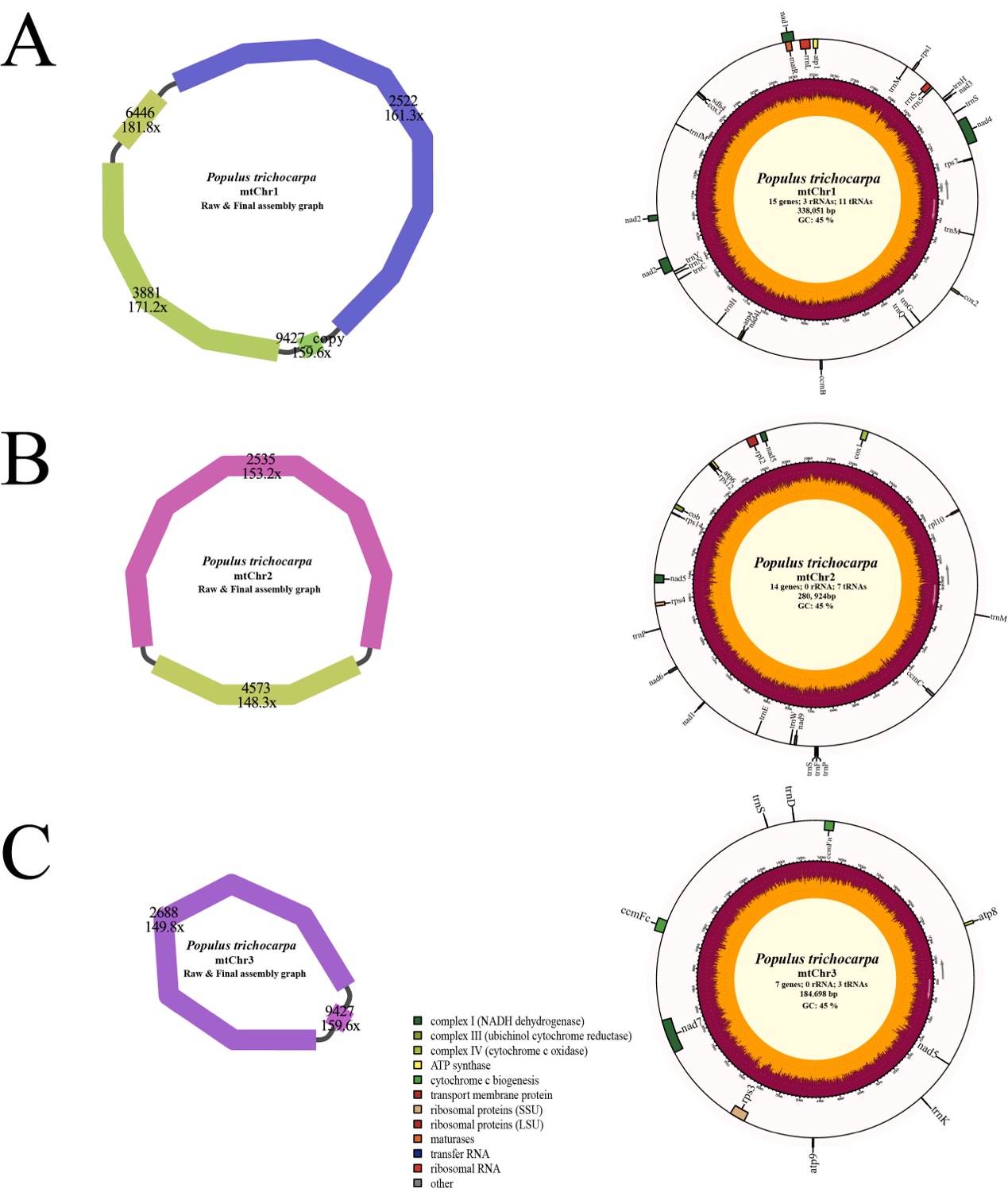


Figure S4. The assembly graphs and genome maps of the *Populus trichocarpa* multi-circular mitogenome.


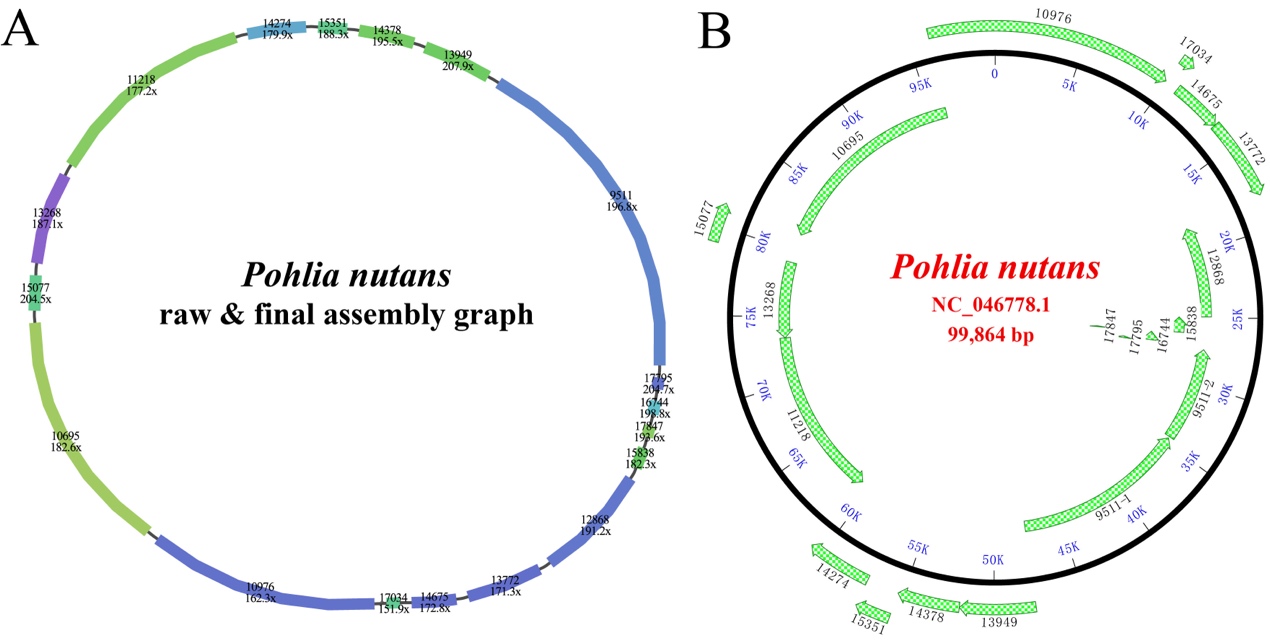


Figure S5. The assembly and alignment graphs of the *Pohlia nutans* mitogenome. The green arrows outside the circle represent contigs resulting from the assembly.


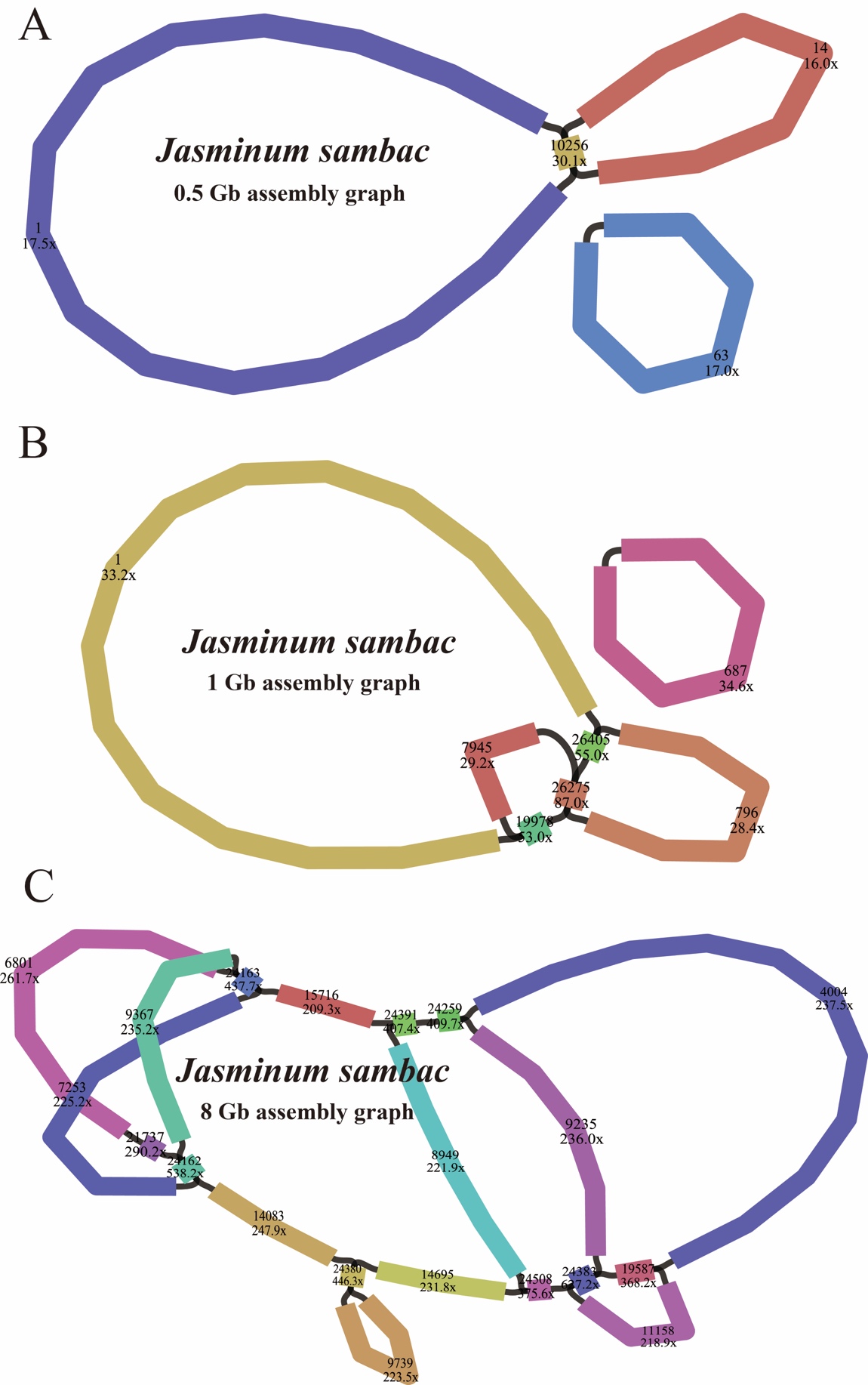


Figure S6. The assembly graphs of *Jasminum sambac* mitogenome. (A) The assembly graph generated from 0.5 Gb HiFi sequencing data. (B) The assembly graph generated from 1 Gb HiFi sequencing data. (C) The assembly graph generated from 8 Gb HiFi sequencing data.

Table S1. Features of all assembled contigs in this study.

| Species | Contig name | Contig length (bp) | Contig depth (×) |
| --- | --- | --- | --- |
| *Pohlia nutans* | 9,511 | 20,170 | 196.8 |
|  | 10,695 | 14,856 | 182.6 |
|  | 10,976 | 13,633 | 162.3 |
|  | 11,218 | 12,665 | 177.2 |
|  | 12,868 | 6,837 | 191.2 |
|  | 13,268 | 5,738 | 187.1 |
|  | 13,772 | 4,537 | 171.3 |
|  | 13,949 | 4,196 | 207.9 |
|  | 14,274 | 3,602 | 179.9 |
|  | 14,378 | 3,433 | 195.5 |
|  | 14,675 | 2,838 | 172.8 |
|  | 15,077 | 2,184 | 204.5 |
|  | 15,351 | 1,788 | 188.3 |
|  | 15,838 | 1,310 | 182.3 |
|  | 16,744 | 853 | 198.8 |
|  | 17,034 | 762 | 151.9 |
|  | 17,795 | 204 | 204.7 |
|  | 17,847 | 127 | 193.6 |
| *Lycopodium japonicum* | 1 | 242,469 | 19.0 |
|  | 2 | 109,765 | 16.1 |
|  | 72 | 67,596 | 15.4 |
|  | 18284* | 16,967 | 35.8 |
|  | 38,422 | 657 | 14.0 |
| *Taxus chinensis* | 1 | 223,165 | 14.3 |
|  | 432 | 137,043 | 14.8 |
|  | 3,297 | 95,550 | 16.5 |
|  | 384986* | 5,127 | 24.0 |
|  | 424,554 | 2,013 | 4.7 |
|  | 448,018 | 943 | 6.1 |
|  | 456,596 | 802 | 10.0 |
| *Juncus effusus* | 406 | 195,956 | 101.2 |
|  | 501 | 138,993 | 96.3 |
|  | 566 | 105,305 | 102.1 |
|  | 831* | 32,408 | 180.2 |
|  | 1,029 | 13,956 | 109.2 |
| *Luzula sylvatica* | 2,888 | 117,796 | 111.4 |
|  | 3,509 | 106,027 | 113.4 |
|  | 5,033 | 82,334 | 109.4 |
|  | 6,596 | 64,917 | 96.7 |
|  | 7,371 | 58,404 | 101.7 |
|  | 10,004 | 41,247 | 100.2 |
|  | 10,291 | 39,416 | 104.8 |
|  | 12,560 | 28,581 | 107.3 |
|  | 16,594 | 15,644 | 75.3 |
|  | 16,757 | 15,301 | 104.1 |
|  | 17,737 | 13,396 | 110.3 |
|  | 17,967 | 12,974 | 99.9 |
|  | 17,999 | 12,927 | 95.3 |
|  | 18,346 | 12,281 | 113.8 |
|  | 21,213 | 7,926 | 102.8 |
|  | 23,121 | 5,754 | 120.5 |
|  | 27583* | 2,419 | 217.3 |
|  | 27691* | 2,356 | 188.1 |
|  | 27843* | 2,264 | 206.7 |
| *Arabidopsis thaliana* | 229 | 60,247 | 39.1 |
|  | 234 | 49,798 | 40.8 |
|  | 236 | 29,699 | 40.6 |
|  | 237 | 50 | 35.0 |
|  | 238 | 19,076 | 45.2 |
|  | 247 | 38,993 | 46.5 |
|  | 255 | 32,171 | 34.9 |
|  | 256 | 31,676 | 34.9 |
|  | 277 | 25,299 | 40.5 |
|  | 315 | 14,679 | 35.8 |
|  | 328 | 12,799 | 36.8 |
|  | 346 | 10,654 | 40.0 |
|  | 388 | 7,174 | 43.8 |
|  | 395* | 6,591 | 78.7 |
|  | 398 | 6,281 | 35.4 |
|  | 402 | 6,167 | 40.7 |
|  | 452* | 4,197 | 75.0 |
|  | 581 | 1,470 | 50.6 |
| *Amaranthus tricolor* | 2,017 | 100,227 | 319.0 |
|  | 2,089 | 94,826 | 262.5 |
|  | 3,447 | 43,134 | 327.5 |
|  | 3,554 | 40,272 | 331.9 |
|  | 3,802 | 34,933 | 311.9 |
|  | 5,087 | 19,375 | 295.7 |
|  | 5,506 | 16,569 | 330.7 |
|  | 5,593 | 15,880 | 329.9 |
|  | 6,577 | 11,315 | 311.0 |
|  | 11,621 | 971 | 4,618.8 |
|  | 11,715 | 902 | 114.2 |
|  | 13,460 | 80 | 2,181.2 |
|  | 13749** | 48 | 974.6 |
| *Helianthus annuus* | 2,103 | 90,599 | 53.1 |
|  | 5,112 | 72,134 | 61.4 |
|  | 13,674 | 51,684 | 43.5 |
|  | 22,226 | 41,966 | 57.5 |
|  | 68,294 | 17,748 | 60.8 |
|  | 76597* | 12,930 | 65.8 |
|  | 106452* | 448 | 108.5 |
| *Jasminum sambac* | 4,004 | 117,285 | 237.5 |
|  | 6,801 | 66,452 | 261.7 |
|  | 7,253 | 60,834 | 225.2 |
|  | 8,949 | 45,739 | 221.9 |
|  | 9,235 | 43,222 | 236.0 |
|  | 9,367 | 42,280 | 235.2 |
|  | 9,739 | 39,645 | 223.5 |
|  | 11,158 | 30,350 | 218.9 |
|  | 14,083 | 17,879 | 247.9 |
|  | 14,695 | 16,258 | 231.8 |
|  | 15,716 | 13,741 | 209.3 |
|  | 19587* | 5,218 | 368.2 |
|  | 21,737 | 2,237 | 290.2 |
|  | 24162* | 271 | 538.2 |
|  | 24163* | 271 | 437.7 |
|  | 24259* | 210 | 409.7 |
|  | 24380* | 135 | 446.3 |
|  | 24383** | 134 | 627.2 |
|  | 24391* | 131 | 407.4 |
|  | 24508* | 67 | 375.6 |
| *Malus domestica* | 1,230 | 193,955 | 158.5 |
|  | 4,733 | 114,892 | 153.4 |
|  | 15,135 | 55,214 | 165.4 |
|  | 35,557 | 13,833 | 171.1 |
|  | 39,125 | 9,793 | 171.0 |
|  | 44882* | 4,631 | 331.7 |
| *Populus trichocarpa* | 2,522 | 199,558 | 161.3 |
|  | 2,535 | 199,269 | 153.2 |
|  | 2,688 | 185,742 | 149.8 |
|  | 3,881 | 113,143 | 171.2 |
|  | 4,573 | 82,527 | 148.3 |
|  | 6,446 | 23,462 | 181.8 |
|  | 9427* | 439 | 319.1 |
| *Salix wilsonii* | 931 | 379,509 | 283.4 |
|  | 1,019 | 331,424 | 266.1 |
|  | 4,249 | 525 | 263.2 |
| *Cuscuta europaea* | 2,607 | 114,116 | 113.1 |
|  | 4,082 | 59,102 | 124.6 |
|  | 5,554 | 39,311 | 115.8 |
|  | 5,634 | 38,649 | 95.7 |
|  | 6,017 | 35,332 | 100.1 |
|  | 6,317 | 32,771 | 119.2 |
|  | 9,603 | 17,082 | 111.9 |
|  | 9,757 | 16,576 | 104.4 |
|  | 10,972 | 12,630 | 112.2 |
|  | 12,617 | 8,070 | 218.5 |
|  | 12,975 | 7,062 | 127.0 |
|  | 13,779 | 4,932 | 107.4 |
|  | 14,133 | 4,229 | 106.2 |
|  | 15,101 | 2,349 | 257.7 |
|  | 15,344 | 1,986 | 265.6 |
|  | 17,713 | 23 | 191.6 |

* Represent the contig containing two copies

** Represent the contig containing three copies

Table S2. Gene compositions and features of 13 mitogenomes.

| Group of genes | Gene name | *Pohlia nutans* | *Lycopodium japonicum* | *Taxus chinensis* | *Juncus effusus* | *Luzula sylvatica* | *Arabidopsis thaliana* | *Amaranthus tricolor* | *Helianthus annuus* | *Jasminum sambac* | *Malus domestica* | *Populus trichocarpa* | *Salix wilsonii* | *Cuscuta europaea* |
| --- | --- | --- | --- | --- | --- | --- | --- | --- | --- | --- | --- | --- | --- | --- |
| ATP synthase | *atp1** | 1 | 1 | 1 | 1 | 1 | 1 | 1 | 1 | 1 | 1 | 1 | 1 | 1 |
|  | *atp4** | 1 | 1 | 1 | 1 | 1 | 1 | 1 | 1 | 1 | 1 | 1 | 1 | 1 |
|  | *atp6** | 1 | 1 | 1 | 1 | 1 | 2 | 1 | 1 | 1 | 1 | 1 | 1 | 1 |
|  | *atp8** | 1 | 0 | 1 | 1 | 1 | 1 | 1 | 1 | 1 | 1 | 1 | 1 | 1 |
|  | *atp9** | 1 | 1 | 1 | 1 | 1 | 1 | 1 | 1 | 1 | 1 | 1 | 1 | 1 |
| Cytochrome c biogenesis | *ccmB** | 1 | 0 | 1 | 1 | 1 | 1 | 1 | 1 | 1 | 1 | 1 | 1 | 1 |
|  | *ccmC** | 1 | 0 | 1 | 1 | 1 | 1 | 1 | 1 | 1 | 1 | 1 | 1 | 1 |
|  | *ccmFc** | 1 | 0 | 1 | 1 | 1 | 1 | 1 | 1 | 1 | 1 | 1 | 1 | 1 |
|  | *ccmFn** | 1 | 0 | 1 | 1 | 1 | 2 | 1 | 2 | 1 | 1 | 1 | 1 | 1 |
| Ubichinol cytochrome c reductase | *cob** | 1 | 1 | 1 | 1 | 1 | 1 | 1 | 1 | 1 | 1 | 1 | 1 | 1 |
| Cytochrome c oxidase | *cox1** | 1 | 1 | 1 | 1 | 1 | 1 | 1 | 1 | 1 | 1 | 1 | 1 | 1 |
|  | *cox2** | 1 | 1 | 1 | 1 | 1 | 1 | 1 | 1 | 1 | 1 | 1 | 1 | 1 |
|  | *cox3** | 1 | 1 | 1 | 1 | 1 | 1 | 1 | 1 | 1 | 1 | 1 | 1 | 1 |
| Maturases | *matR** | 0 | 0 | 1 | 1 | 1 | 1 | 1 | 1 | 1 | 1 | 1 | 1 | 1 |
| Transport membrane protein | *mttB** | 1 | 1 | 1 | 1 | 1 | 1 | 1 | 1 | 1 | 1 | 1 | 1 | 1 |
| NADH dehydrogenase | *nad1** | 1 | 1 | 1 | 1 | 1 | 1 | 1 | 1 | 1 | 1 | 1 | 1 | 1 |
|  | *nad2** | 1 | 1 | 1 | 1 | 1 | 1 | 1 | 1 | 1 | 1 | 1 | 1 | 1 |
|  | *nad3** | 1 | 1 | 1 | 1 | 1 | 1 | 1 | 1 | 1 | 1 | 1 | 1 | 1 |
|  | *nad4** | 1 | 1 | 1 | 1 | 1 | 1 | 1 | 1 | 1 | 1 | 1 | 1 | 1 |
|  | *nad4L** | 1 | 1 | 1 | 1 | 1 | 1 | 1 | 1 | 1 | 1 | 1 | 1 | 1 |
|  | *nad5** | 1 | 1 | 1 | 2 | 1 | 1 | 1 | 1 | 1 | 1 | 1 | 1 | 1 |
|  | *nad6** | 1 | 1 | 1 | 1 | 1 | 1 | 1 | 1 | 1 | 1 | 1 | 1 | 1 |
|  | *nad7** | 0 | 0 | 1 | 1 | 1 | 1 | 1 | 1 | 1 | 1 | 1 | 1 | 1 |
|  | *nad9** | 1 | 1 | 1 | 1 | 1 | 1 | 1 | 1 | 1 | 1 | 1 | 1 | 1 |
| Ribosomal proteins (LSU) | *rpl2* | 1 | 1 | 0 | 0 | 0 | 1 | 0 | 0 | 2 | 0 | 1 | 1 | 0 |
|  | *rpl5* | 1 | 1 | 1 | 1 | 1 | 1 | 1 | 1 | 1 | 1 | 0 | 0 | 1 |
|  | *rpl6* | 1 | 1 | 0 | 0 | 0 | 0 | 0 | 0 | 0 | 0 | 0 | 0 | 0 |
|  | *rpl10* | 1 | 1 | 0 | 0 | 0 | 0 | 0 | 1 | 1 | 0 | 1 | 1 | 1 |
|  | *rpl16* | 1 | 1 | 1 | 1 | 1 | 1 | 0 | 1 | 1 | 0 | 1 | 1 | 1 |
| Ribosomal proteins (SSU) | *rps1* | 1 | 0 | 0 | 0 | 0 | 0 | 0 | 0 | 0 | 1 | 1 | 1 | 1 |
|  | *rps2* | 1 | 1 | 0 | 1 | 1 | 0 | 0 | 0 | 0 | 0 | 0 | 0 | 0 |
|  | *rps3* | 1 | 1 | 1 | 1 | 1 | 1 | 1 | 1 | 1 | 1 | 1 | 1 | 1 |
|  | *rps4* | 1 | 1 | 1 | 1 | 1 | 1 | 1 | 1 | 1 | 1 | 1 | 1 | 1 |
|  | *rps7* | 1 | 0 | 0 | 1 | 1 | 1 | 1 | 0 | 1 | 0 | 1 | 0 | 0 |
|  | *rps10* | 0 | 1 | 0 | 1 | 1 | 0 | 0 | 0 | 2 | 0 | 0 | 0 | 1 |
|  | *rps11* | 1 | 1 | 0 | 0 | 0 | 0 | 0 | 0 | 0 | 0 | 0 | 0 | 0 |
|  | *rps12* | 0 | 1 | 1 | 1 | 1 | 1 | 1 | 1 | 1 | 1 | 1 | 1 | 1 |
|  | *rps13* | 1 | 1 | 1 | 1 | 1 | 0 | 1 | 1 | 1 | 1 | 0 | 0 | 1 |
|  | *rps14* | 1 | 1 | 0 | 1 | 1 | 1 | 0 | 0 | 1 | 1 | 1 | 0 | 0 |
|  | *rps19* | 1 | 1 | 1 | 1 | 1 | 2 | 0 | 0 | 0 | 1 | 0 | 0 | 1 |
| Succinate dehydrogenase | *sdh3* | 1 | 1 | 0 | 0 | 0 | 0 | 0 | 0 | 1 | 1 | 0 | 0 | 0 |
|  | *sdh4* | 1 | 1 | 1 | 0 | 0 | 1 | 0 | 0 | 1 | 1 | 1 | 1 | 1 |
| Ribosomal RNAs | *rrn5* | 1 | 2 | 2 | 1 | 1 | 1 | 1 | 1 | 1 | 1 | 1 | 1 | 1 |
|  | *rrn18* | 1 | 2 | 2 | 1 | 1 | 1 | 1 | 1 | 1 | 1 | 1 | 1 | 1 |
|  | *rrn26* | 1 | 2 | 1 | 1 | 1 | 1 | 1 | 1 | 1 | 2 | 1 | 1 | 1 |
| Transfer RNAs | *trnA* | 1 | 1 | 0 | 0 | 0 | 0 | 0 | 0 | 0 | 0 | 0 | 0 | 0 |
|  | *trnC* | 1 | 1 | 0 | 1 | 1 | 1 | 2 | 1 | 1 | 1 | 1 | 1 | 1 |
|  | *trnD* | 1 | 1 | 2 | 1 | 1 | 1 | 1 | 1 | 1 | 1 | 1 | 1 | 1 |
|  | *trnE* | 1 | 1 | 2 | 1 | 1 | 1 | 1 | 1 | 1 | 1 | 1 | 1 | 1 |
|  | *trnF* | 1 | 1 | 0 | 1 | 1 | 4 | 1 | 1 | 1 | 3 | 1 | 1 | 2 |
|  | *trnG* | 2 | 3 | 0 | 0 | 0 | 1 | 1 | 1 | 1 | 1 | 1 | 1 | 1 |
|  | *trnH* | 1 | 2 | 0 | 1 | 1 | 1 | 1 | 1 | 1 | 1 | 2 | 2 | 1 |
|  | *trnK* | 1 | 2 | 0 | 1 | 1 | 1 | 1 | 2 | 1 | 1 | 1 | 1 | 1 |
|  | *trnL* | 2 | 5 | 0 | 0 | 0 | 0 | 0 | 0 | 0 | 0 | 0 | 0 | 0 |
|  | *trnM* | 3 | 6 | 3 | 4 | 3 | 4 | 3 | 5 | 5 | 4 | 4 | 4 | 6 |
|  | *trnN* | 0 | 2 | 0 | 1 | 1 | 1 | 1 | 1 | 1 | 1 | 1 | 1 | 1 |
|  | *trnP* | 1 | 2 | 0 | 2 | 1 | 1 | 2 | 1 | 1 | 2 | 1 | 1 | 1 |
|  | *trnQ* | 1 | 2 | 0 | 1 | 1 | 1 | 1 | 1 | 1 | 1 | 1 | 1 | 1 |
|  | *trnR* | 2 | 4 | 1 | 0 | 0 | 1 | 0 | 0 | 0 | 0 | 0 | 0 | 0 |
|  | *trnS* | 1 | 3 | 0 | 3 | 2 | 3 | 2 | 1 | 3 | 1 | 3 | 3 | 3 |
|  | *trnT* | 1 | 0 | 0 | 0 | 0 | 0 | 0 | 0 | 0 | 0 | 0 | 0 | 0 |
|  | *trnV* | 1 | 1 | 0 | 0 | 0 | 0 | 0 | 1 | 0 | 0 | 0 | 1 | 0 |
|  | *trnW* | 1 | 1 | 1 | 1 | 1 | 1 | 1 | 1 | 1 | 1 | 1 | 1 | 1 |
|  | *trnY* | 1 | 2 | 1 | 1 | 1 | 2 | 1 | 1 | 1 | 1 | 1 | 1 | 1 |

*Plant mitochondrial core gene

Table S3. Comparison of the mitogenome assembly between PMAT and GSAT

| Species | Contig number | | Mitogenome size (bp) | | Number of Variations | Similarity (%) |
| --- | --- | --- | --- | --- | --- | --- |
|  | PMAT | GSAT | PMAT | GSAT |  |  |
| *Populus trichocarpa*^a^ | 8 | 5 | 804,579 | 804,486 | 93 | 99.9884 |
| *Malus domestica*^b^ | 6 | 6 | 396,949 | 397,677 | 728 | 99.8169 |

^a^Represent the sequencing data generated from the same sample

^b^Represent the sequencing data generated from different samples

Table S4. The benchmarking results of 13 mitogenome assemblies using Hifiasm with 20 CPUs.

| Organism | Data (Gb) | No. of total contigs | No. of mt-like contigs (>10 kb) | Assembled mitogenome size (bp) | Asembly completed | Mitogenome structure | Elapsed time (Min) | Memory usage (GB) |
| --- | --- | --- | --- | --- | --- | --- | --- | --- |
| *Pohlia nutans* | 31.16 | 1,698 | 4 | - | NO | - | 4,984.9 | 38.32 |
| *Lycopodium japonicum* | 4.04 | 7,657 | 1 | 454,420 | YES | One circle | 34.2 | 24.85 |
| *Taxus chinensis* | 17.66 | 38,580 | 1 | 469,792 | YES | One circle | 634.1 | 49.1 |
| *Juncus effusus* | 22.56 | 639 | 10 | - | NO | - | 2,746.3 | 48.6 |
| *Luzula sylvatica* | 4.74 | 3,851 | 9 | - | NO | - | 584.8 | 20.79 |
| *Arabidopsis thaliana* | 22.9 | 1,507 | 11 | - | NO | - | 3,415.9 | 50.85 |
| *Amaranthus tricolor* | 25.09 | 2,830 | 14 | 382,383 | YES | Four circles | 1,978.7 | 32.24 |
| *Cuscuta europaea* | 25.97 | 477 | 14 | - | NO | - | 1,638.5 | 38.83 |
| *Helianthus annuus* | 12.82 | 31,348 | 39 | - | NO | - | 1,240.5 | 60.24 |
| *Jasminum sambac* | 43.2 | 444 | 14 | - | NO | - | 3,779.5 | 62.89 |
| *Malus domestica* | 9.39 | 8,348 | 108 | - | NO | - | 265.5 | 24.88 |
| *Populus trichocarpa* | 6.96 | 862 | 3 | 803,750 | YES | Three circles | 186.1 | 20.65 |
| *Salix wilsonii* | 10.26 | 114 | 1 | 711,456 | YES | One circle | 513.6 | 20.91 |
